# A Biosynthetic Platform for Antimalarial Drug Discovery

**DOI:** 10.1128/AAC.02129-19

**Published:** 2020-04-21

**Authors:** Mark D. Wilkinson, Hung-En Lai, Paul S. Freemont, Jake Baum

**Affiliations:** a Department of Chemistry, Imperial College London, London, United Kingdom; b Department of Infectious Diseases, Faculty of Medicine, Imperial College London, London, United Kingdom; c Department of Life Sciences, Imperial College London, London, United Kingdom; d UK Dementia Research Institute Care Research and Technology Centre, Imperial College London, United Kingdom

**Keywords:** violacein, drug discovery, antimalarial, drug resistance, synthetic biology

## Abstract

Advances in synthetic biology have enabled the production of a variety of compounds using bacteria as a vehicle for complex compound biosynthesis. Violacein, a naturally occurring indole pigment with antibiotic properties, can be biosynthetically engineered in Escherichia coli expressing its nonnative synthesis pathway. To explore whether this synthetic biosynthesis platform could be used for drug discovery, here we have screened bacterially derived violacein against the main causative agent of human malaria, Plasmodium falciparum. We show the antiparasitic activity of bacterially derived violacein against the P. falciparum 3D7 laboratory reference strain as well as drug-sensitive and -resistant patient isolates, confirming the potential utility of this drug as an antimalarial agent. We then screen a biosynthetic series of violacein derivatives against P. falciparum growth. The varied activity of each derivative against asexual parasite growth points to the need to further develop violacein as an antimalarial. Towards defining its mode of action, we show that biosynthetic violacein affects the parasite actin cytoskeleton, resulting in an accumulation of actin signal that is independent of actin polymerization. This activity points to a target that modulates actin behavior in the cell either in terms of its regulation or its folding. More broadly, our data show that bacterial synthetic biosynthesis could become a suitable platform for antimalarial drug discovery, with potential applications in future high-throughput drug screening with otherwise chemically intractable natural products.

## Abstract (precis)

Advances in synthetic biology have enabled the production of a variety of compounds using bacteria as a vehicle for complex compound biosynthesis. Violacein, a naturally occurring indole pigment with antibiotic properties, can be biosynthetically engineered in Escherichia coli expressing its nonnative synthesis pathway. To explore whether this synthetic biosynthesis platform could be used for drug discovery, here we have screened bacterially derived violacein against the main causative agent of human malaria, Plasmodium falciparum.

## INTRODUCTION

Malaria has a huge global health burden, with around half of the world’s population at risk of contracting the disease which killed over 400,000 people in 2017 (1). Malaria disease is caused by apicomplexan parasites from the genus *Plasmodium*, with Plasmodium falciparum causing the majority of deaths worldwide. The symptoms of malaria disease develop during the asexual stages of the parasite life cycle, which occurs in the bloodstream. Here, the parasite undergoes multiple rounds of growth, replication, and invasion of red blood cells. Various drugs have been developed to target the asexual stages of the parasite, but, inevitably, resistance has evolved to every major front-line therapy for malaria treatment, including, most recently, artemisinin combined therapies (ACTs) (2). Multidrug resistance to ACTs, focused in the Greater Mekong Subregion of South East Asia, has been reported both as delayed parasite clearance and, more worryingly, treatment failure (3). The challenges of emerging drug resistance combined with the cost associated with the development of new drugs make it essential to explore new ways to develop novel antimalarial compounds.

**FIG 1:**
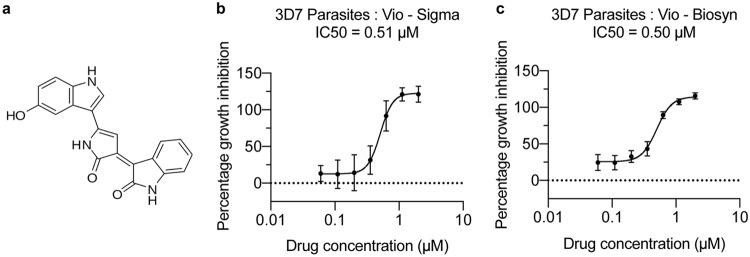
Plasmodium falciparum asexual growth inhibition assays with violacein. (a) The chemical structure of violacein (PubChem CID 11053). (b, c) Commercially available violacein (b) and biosynthetic violacein (c) kill asexual 3D7 parasites with a 50% inhibitory concentration of 0.51 μM (Vio-Sigma) and 0.50 μM (Vio-Biosyn).

Previous work identified violacein, a violet indolocarbazole pigment produced by bacteria (Fig. 1a), as a potential antimalarial agent able to kill both asexual P. falciparum parasites *in vitro* and protect against malaria infection in a mouse malaria model *in vivo* (4–6). Violacein’s antimalarial activity has, therefore, identified it as a potential agent for future drug development. However, commercial violacein samples can only be obtained through laborious purification from bacteria (*Chromobacterium* sp. [7, 8] or *Janthinobacterium* sp. [9]) because of the complexity of its highly aromatic structure (Fig. 1a). Purification from these bacteria requires specialized equipment and high-level biosafety equipment since these bacteria themselves can cause deadly infections (10). As such, commercially available violacein is extremely expensive. Alternative strategies of violacein synthesis are being explored, in particular, the use of synthetic biology to engineer industrial bacterial species that can express nonnative violacein. Several groups, including ours (11), have been successful in implementing a five-gene violacein biosynthetic pathway (vioABCDE) into Escherichia coli or other heterologous hosts (12–14), providing a route for robust, in-house, and inexpensive compound production.

We have previously extended the success of this biosynthetic pathway by generating combinations of 68 new violacein and deoxyviolacein analogs. These combinations are achieved by feeding various tryptophan substrates to recombinant E. coli expressing the violacein biosynthetic pathway or via introduction of an *in vivo* chlorination step—the tryptophan 7-halogenase RebH from the rebeccamycin biosynthetic pathway (13, 15–17). This biosynthetic approach is able to produce large quantities of compound derivatives using simple, inexpensive, and nonhazardous bacteria compared with native-producing strains in a sustainable and flexible approach.

Here, we set out to explore whether the use of this biosynthetic system could be developed as a route to antimalarial compound production and testing by measuring the activity of derivatives on the growth of P. falciparum sexual and asexual parasites. We have confirmed the viability of the system, ensuring there is no background antiparasitic activity in bacterial solvent extracts lacking violacein. We then tested the biosynthetic violacein extract from E. coli and confirmed its 50% inhibitory concentration (IC_50_), which is in agreement with a commercial violacein standard and previous studies (14). Finally, as well as using this approach to explore the mode of action of violacein, we show that extracts representing a diverse series of biosynthetically derived variants show various effects on parasite growth, with 16 of the 28 compound mixtures inhibiting growth to a greater level than the parent violacein molecule. Indeed, one purified compound, 7-chloroviolacein, exhibits an ∼20% higher inhibition activity than the underivatized violacein compound. The screening approach used in this study suggests that biosynthetic systems may, therefore, provide an, as yet, untapped resource for screening complex compounds and optimizing them for antimalarial discovery.

## RESULTS

### Violacein expressed using synthetic operons kills P. falciparum 3D7 parasites.

Previous work has shown that violacein is able to kill asexual *Plasmodium* parasites *in vitro* and in a mouse model *in vivo* (9, 13). Violacein cytotoxicity is highly dependent on cell type, ranging from an IC_50_ value of around 2.5 μM in HepG2 cells to up to 12 μM in fibroblasts and potential erythrocytic rupture at concentrations above 10 μM (13, 18). Taking this into consideration, we used concentrations of 2 μM or less of violacein to explore the growth inhibition of P. falciparum asexual stages, noting no phenotypic effect on erythrocyte morphology at the highest final concentration (see Fig. S1 in the supplemental material). Our biosynthetic system for violacein production requires chloramphenicol drug pressure, which is known to affect parasite viability (19). We first set out to ensure that presence of this known antibiotic did not affect parasite growth. Extract from bacteria lacking the violacein-producing enzymes but grown under chloramphenicol pressure (i.e., background) did not affect parasite viability (see Fig. S2 in the supplemental material). This gave us confidence that extracts from biosynthetically modified E. coli would report only on the activity of a drug produced but not from background chloramphenicol contamination. To test this, we compared the activity of a commercial violacein standard (Vio-Sigma) with violacein derived from bacterial solvent extracts from E. coli biosynthesis (Vio-Biosyn) on wild-type P. falciparum 3D7 growth, using a well-established asexual growth inhibition assay. No difference in the IC_50_ values between the two violacein samples was seen (Fig. 1b and c). We further tested the two violacein samples on sexual parasites by measuring exflagellation (20) and saw no difference in the IC_50_ values of around 1.7 μM (see Fig. S3 in the supplemental material), but complete IC_50_ curves could not be generated without going above the cytotoxic threshold of 2.5 μM. These data demonstrate that solvent-extracted violacein from E. coli (Vio-Biosyn) is active and that its production provides a suitable platform for developing and testing potential antimalarial compounds.

### Biosynthetic violacein extract kills both artemisinin-resistant and -sensitive field isolate parasites.

**FIG 2:**
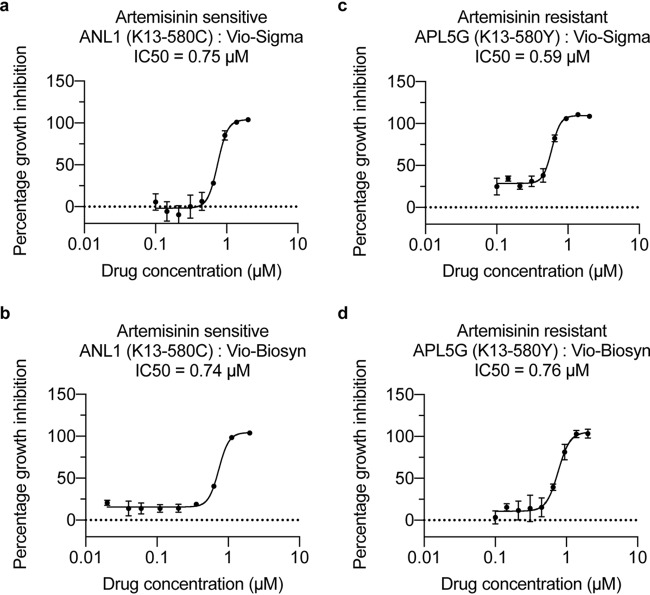
Biosynthetic violacein kills both artemisinin-sensitive and -resistant parasite clinical isolates. (a to d) Both commercially available violacein and biosynthetic violacein are able to kill parasite clinical isolates either sensitive (ANL1/Kelch13 wild-type) (a, b) or resistant to artemisinin (APL5G/Kelch13 C580Y) (c, d).

To explore whether violacein has utility for addressing emerging ACT drug resistance in the field, we tested the efficacy of Vio-Sigma and Vio-Biosyn on two parasite clinical isolates derived from the Greater Mekong Subregion, where ACT resistance is concentrated. Both clinical isolates have been phenotypically characterized in the clinic setting, showing either treatment failure or success, adapted to culture, and genotyped for the C580Y Kelch-13 resistance marker (21) that is known to correlate with sensitivity to artemisinin-based drugs. Both the artemisinin-sensitive isolate (ANL1; Kelch13 wild-type) and the artemisinin-resistant isolate (APL5G; Kelch13 C580Y) were sensitive to Vio-Biosyn and Vio-Sigma, with similar IC_50_ values (Fig. 2a to d). Activity against artemisinin resistance provides support for the development of the violacein scaffold to address emerging drug resistance in the field.

### Violacein derivatives show potent antimalarial activity.

**FIG 3:**
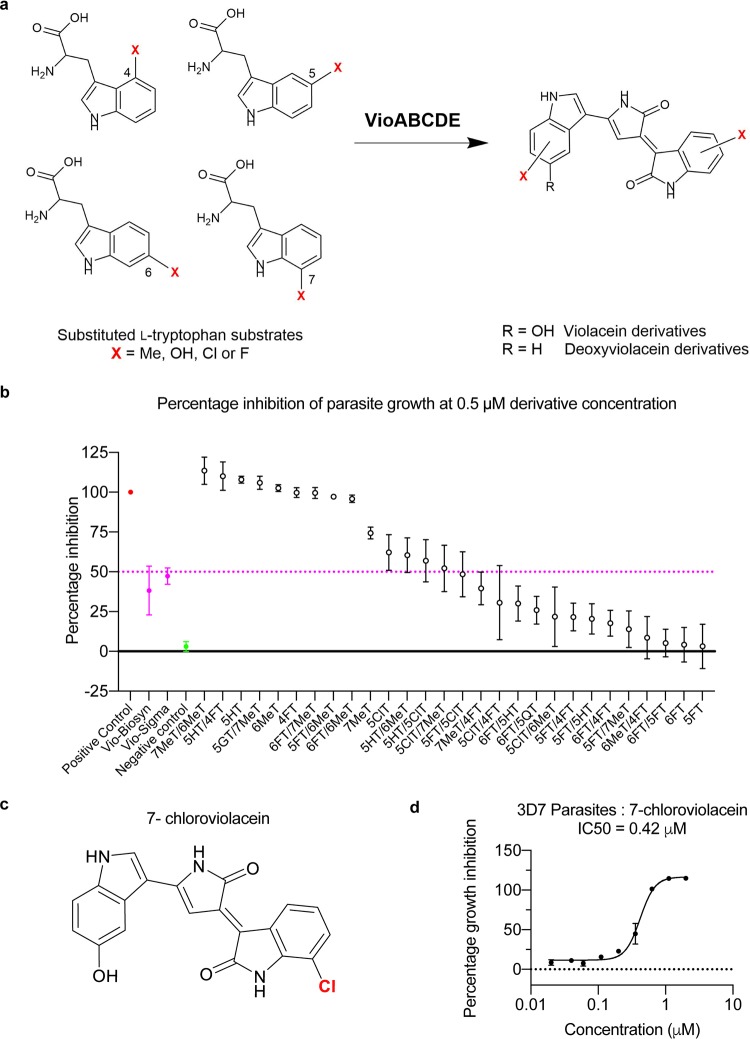
Plasmodium falciparum growth inhibition assays testing a series of biosynthetic violacein derivatives. (a) Tryptophan derivatives (left) used to generate violacein derivatives (right). (b) A screen of violacein derivative mixtures at 0.50 μM (adjusted by measuring absorbance at 575 nm) shows 8 mixtures with >95% inhibition of parasite growth, while 12 mixtures are less potent than Vio-Biosyn. (c) The chemical structure of the purified derivative 7-chloroviolacein. (d) The activity of the purified violacein derivative 7-chloroviolacein shows an IC_50_ value of 0.42 μM against 3D7 wild-type parasites.

To explore whether bacterial biosynthesis could be used further to generate compound derivatives, increasing the throughput of complex molecule testing, we obtained extracts from 28 bacterial strains, each modulated to synthesize a mixture of violacein analogues (Fig. 3a; see Fig. S4 in the supplemental material) (22). The bacterial extracts were produced by feeding corresponding tryptophan substrates as described previously (14), and violacein concentrations in the extracts were calibrated against a violacein standard. Asexual growth assays were, again, carried out by testing each extract at the IC_50_ of biosynthetic violacein, 0.50 μM. We saw a large variation of inhibition of parasite growth, with 8 compound mixtures exhibiting >95% inhibition, while 12 others showed a decreased effect on parasite growth (Fig. 3b). As a proof-of-concept, one of the more active extracts was used to purify the violacein derivative 7-chloroviolacein (Fig. 3c). 7-chloroviolacein exhibited an IC_50_ value of 0.42 μM. This purified derivative is at least equipotent to the parent violacein compound (Fig. 3d). Given the speed and low cost of extracting these violacein analogs and purifying them directly from bacteria, these data, therefore, suggest an entirely new approach to complex compound drug testing for antimalarial discovery and optimization.

### Biosynthetic violacein affects actin dynamics in the cell but does not affect polymerization *in vitro*.

The mode of action of violacein against P. falciparum parasites has not previously been characterized. The treatment of a variety of human-derived cell lines with violacein show a range of responses, with one patient-derived glioblastoma cell line having compromised motility and increased rounding up, attributed to a disruption of the filamentous actin network (23). Towards exploring the phenotype associated with its activity, we performed flow cytometry and immunofluorescence assays (IFAs) to observe any changes in the parasite under biosynthetic violacein treatment. A 3D7-derived parasite line expressing a constitutive cytoplasmic green fluorescent protein (sfGFP) marker was labeled with DNA marker 4′,6-diamidino-2-phenylindole (DAPI) and a monoclonal antibody that preferentially recognizes filamentous actin (6, 24) to explore overall parasite morphology in the cytoplasm, nucleus, and actin cytoskeleton, respectively. Parasites were then treated with either a negative control, dimethyl sulfoxide (DMSO), or a positive control, actin filament stabilizing compound jasplakinolide (JAS), as well as Vio-Biosyn. Parasites were checked by flow cytometry for any differences in overall signal (see Fig. S5 in the supplemental material). A low actin-positive signal was seen with DMSO treatment, as expected given the predominance of short, transient filaments and globular actin in asexual parasites (25). However, the intensity of actin labeling following JAS and violacein treatment both showed marked increases compared with that of DMSO (Fig. S5).

**FIG 4:**
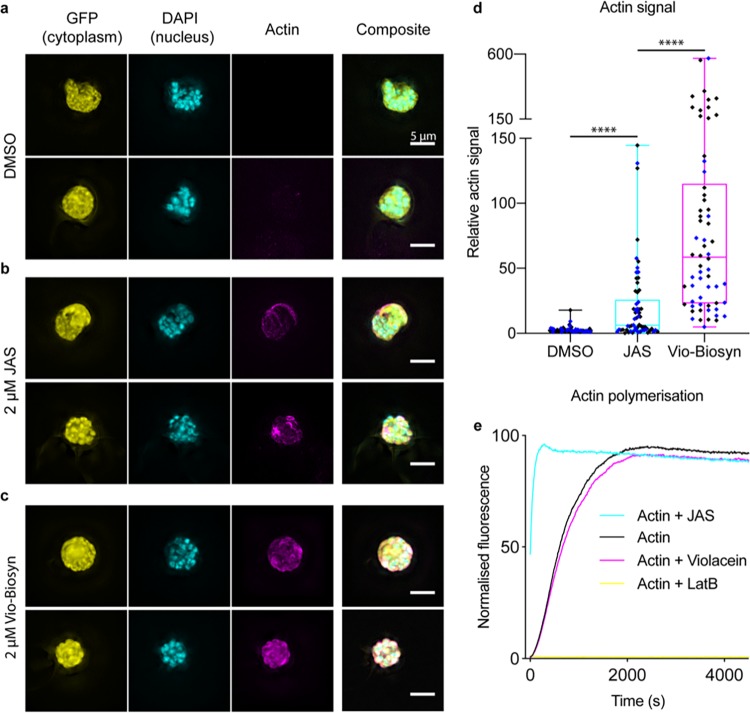
The phenotype of biosynthetic violacein treatment on cytoplasmic GFP expressing 3D7 parasites suggests modulation of the actin cytoskeleton through indirect action. (a) 3D7 parasites expressing cytoplasmic GFP and treated with DMSO have a diffuse actin signal. (b) Parasites treated with 2 μM jasplakinolide (JAS), which stabilizes filament formation, have clearly formed actin filament structures that localize to the parasite cell periphery. (c) Parasites treated with 2 μM biosynthetic violacein have increased local actin concentrations both around the outside the cell and in the center. (d) Parasites treated with biosynthetic violacein have a much greater overall actin signal than both DMSO control and JAS-treated cells, as measured by actin fluorescence relative to cytoplasmic GFP signal. Data shown are of 62 images over two biological repeats (black and blue) for each sample. *P* values are from an unpaired Student’s *t* test; ****, *P* < 0.0001. (e) Biosynthetic violacein shows no effect on actin polymerization *in vitro*, as measured by pyrene-actin polymerization, compared with two known actin binders (latrunculin B, which binds to the monomer and prevents filament formation, and jasplakinolide, which increases nucleation and stabilizes actin filaments). LatB, latrunculin B; JAS, jasplakinolide. Scale bar = 5 μm.

To explore the nature of flow cytometry changes in actin intensity following violacein treatment, IFAs were undertaken. In the DMSO-treated parasites, the GFP signal is spread throughout the cytoplasm along with a clearly defined nucleus, as expected (Fig. 4a). The actin signal is diffuse with a low background (Fig. 4a). Following JAS treatment, actin filaments are known to be stabilized (25), producing an expected concentrated overall actin signal (Fig. 4b), indicative of high local concentrations of polymerized actin. Parasites treated with Vio-Biosyn also gave a much higher actin signal than untreated controls, although it was distinct from that following JAS treatment (Fig. 4c). The concentrated signal from Vio-Biosyn was broader across the cell and less focused in localized regions of the cell periphery. This matched the overall intensity of signal seen by flow cytometry and relative to sfGFP signal as a control for parasite size (Fig. 4d). In the DMSO-treated control, the diffuse actin signal is 3% of the total GFP signal. This increases to 27% upon JAS treatment, which is indicative of an increased number of filaments, whereas parasites treated with Vio-Biosyn reach a mean average signal 98%, representing a huge increase in actin accumulation in the cell. This broad concentration of actin signal would be indicative of either massively increased filament nucleation or actin aggregation, as caused by actin misfolding. To test whether Vio-Biosyn directly affects actin filament formation (as JAS does), both drugs were assayed using a pyrene-labeled actin assembly assay that was used previously to test compound derivatives for actin activity (25). No effect on actin polymerization was seen with Vio-Biosyn compared with either JAS (filament nucleating) or the monomer-stabilizing drug latrunculin B (Fig. 4e). Together, these observations suggest Vio-Biosyn does not directly interact with actin. It is, therefore, possible that Vio-Biosyn interacts with actin indirectly, such as via the known actin-binding partners in the parasite cell (26) or via an alternative pathway involved in actin folding, which would give rise to actin aggregation within the cell.

## DISCUSSION

The emergence of resistance to front-line artemisinin-based drug treatments for malaria is a major threat to global health. As such, new antimalarial treatments are in urgent demand. Here, we tested violacein, a compound with known antibacterial, antitumorigenic, and antiparasitic activity, against P. falciparum parasites, validating its potential utility for antimalarial drug development. We showed that biosynthetically produced violacein was as effective as commercially available violacein, with a mode of action that affects the actin cytoskeleton of the parasite. We also successfully tested 28 violacein analog mixtures using a high-throughput growth assay on asexual parasites, suggesting this method of biosynthetic production is a suitable platform for antimalarial discovery and optimization.

Previous work has shown that violacein is capable of killing lab-derived chloroquine-resistant P. falciparum parasites (14). Here, we showed that patient-sourced clinical isolates, sensitive or resistant to artemisinin, could equally be killed by both commercial and biosynthetic violacein, with similar IC_50_ values. Our results show violacein inhibits asexual parasite growth with an IC_50_ value of at least an order of magnitude more potent than fibroblasts and lymphocytes found in circulation in the blood (0.5 μM versus >10 μM) (13, 18). Furthermore, although full IC_50_ curves could not be generated, the compounds both inhibit development of the sexual stages of the parasite life cycle, with an IC_50_ of around 1.7 μM (Fig. S3). Any compound identified using this assay with an IC_50_ of less than 2.0 μM is considered for further compound development (27). Combined, these data suggest violacein is a potential drug that could be developed to antagonize resistance in the field and target both asexual- and sexual-stage parasites. While the derivative library tested consisted of mixtures of violacein analogs, it is encouraging to see some of these compound mixtures have considerably more potent antimalarial activity than violacein itself. Critically, when we tested a purified compound (7-chloroviolacein), we saw at least equipotent activity of the derivative compound, illustrating the potential of biosynthetic production of antimalarial compounds for rapid screening and rational drug optimization.

Interestingly, violacein-treated parasites have cytoskeletal deformities that suggested a disruption to actin modulation within the parasite. Given violacein has no effect on actin polymerization kinetics *in vitro*, it is possible that the phenotype observed is as a result of actin aggregation in the cell, which could be a side effect of actin misfolding. P. falciparum requires actin as an essential part of its motor complex and for other processes in the cell (9). Unlike most proteins, actin requires a dedicated chaperonin system to fold into its native state (28). Of note, this entire pathway is highly upregulated in artemisinin-resistant parasite isolates (29) and would constitute a well-justified target for antagonizing drug resistance in the field. Further work in testing the effects of violacein on actin folding or modulation are clearly required to explore whether this is the target for the drug. Ultimately, the ability of violacein to affect such a major pathway as actin dynamics in the cell, as well as kill drug-resistant parasites, provides an encouraging outlook for its therapeutic development.

In summary, our data show that a bacterial biosynthetic platform for creating compounds and their derivatives is suitable for testing for antimalarial drug development. As the need for novel therapeutics increases and the interest in natural compounds, often complex in nature, grows, we hope to use this approach to develop novel chemical scaffolds in a high-throughput manner toward finding the next generation of antimalarials.

## MATERIALS AND METHODS

### Generation and extraction of violacein and derivatives.

Violacein and derivatives were generated and extracted as previously described (30). Briefly, E. coli JM109 strain (Promega) was transformed with the violacein pathway plasmid [pTU2S(VioAE)-b-VioBCD] and grown overnight before being inoculated into LB broth until the optical density at 600 nm (OD_600_) reached 0.5. These cultures were then supplemented with either tryptophan or a synthetic tryptophan analog at 0.04% (wt/vol) and grown at 25°C for up to 65 h before pelleted at 4,000 rpm. The cell pellet was then resuspended with 1/10th volume of ethanol to extract violacein, followed by centrifugation to separate the ethanol supernatant containing violacein extract from cell debris. The supernatant was then dried *in vacuo* and stored at –20°C for long-term storage or reconstituted in DMSO for growth inhibition assays. Concentrations of violacein in the bacterial solvent extracts were calibrated against a commercial violacein standard (Sigma) based on absorbance at 575 nm. Compound mixtures used in the growth inhibition assay consist of mixtures of violacein derivatives (Fig. S4), as described previously (14).

### Plasmodium falciparum growth inhibition assays.

P. falciparum parasite lines 3D7, ANL1, and APL5G were used for the growth inhibition assays (GIAs). A 3D7 sfGFP line was used for immunofluorescence assays (31). All parasite lines were cultured in complete RPMI (RPMI-HEPES culture media [Sigma] supplemented with 0.05 g/liter hypoxanthine, 0.025 g/liter gentamicin, and 5 g/liter Albumax II [ThermoScientific]) and maintained at 1% to 5% parasitemia and 4% hematocrit. For the GIA, 96-well plates were predispensed with a serial dilution of the compound and normalized to 1% DMSO. Double-synchronized ring-stage parasites (1% parasitemia, 2% hematocrit, complete medium, and sorbitol synchronized at ring stage at 0 h and 48 h) were added to the wells to a total volume of 101 μl. Cultures were incubated for 72 h at 37°C in a gas mixture of 90% N_2_, 5% O_2_, and 5% CO_2_. Red blood cells were lysed through a freeze-thaw step at –20°C, and parasites were resuspended and lysed with 100 μl lysis buffer (20 mM Tris-HCl [pH 7.5], 5 mM EDTA, 0.008% saponin, and 0.8% Triton X-100) containing 0.2% SYBR green and incubated for 1 h at room temperature. SYBR green fluorescence (excitation, 485 nm; emission, 535 nm) was measured using a Tecan infinite M200 Pro instrument. Data shown are the mean average of 3 biological replicates (± SEM), each of which is a mean average of 3 technical replicates (unless stated otherwise), and is normalized to a positive control (cycloheximide) and a negative control (DMSO only). IC_50_ values were calculated using GraphPad Prism version 8.0.

### Immunofluorescence assays.

A total of 100 μl of a mixed-stage sfGFP parasite line (5% parasitemia and 2% hematocrit) was incubated for 24 h with 2 μM the compound of interest. At 0 h and 6 h, parasites were fixed with 4% paraformaldehyde and 2% glutaraldehyde (Electron Microscopy Sciences) and incubated on a roller for 20 min at room temperature (RT), before being pelleted at 3,000 rpm and washed three times in 100 μl 1× phosphate-buffered saline (PBS). The cells were subsequently permeabilized in 0.1% Triton X-100 (Sigma) for 10 min at RT before being pelleted and washed three times in PBS as before. Cells were blocked in 3% bovine serum albumin (BSA) in PBS for 1 h at RT on a roller before being incubated with a primary antibody (1:500 mouse anti-actin 5H3 [14]) for 1 h at RT. Cells were washed three times with PBS before the addition of the secondary antibody (1:1,000 anti-mouse Alexa 647 conjugated) for 1 h at RT. Cells were washed three times in PBS and resuspended in 100 μl PBS with 0.05% DAPI. Cells were diluted 30-fold and loaded onto polylysine-coated coverslips (ibidi) before being imaged. Imaging was performed on a Nikon Ti-E microscope using a 100× Plan Apo 1.4 NA oil lens objective with DAPI, fluorescein isothiocyanate (FITC), and Cy5 specific filter sets. Image stacks were captured 3 μm on either side of the focal plane, with a z-step of 0.2 μm. Image analysis was conducted on raw image data sets in ImageJ, calculating a ratio between Alexa Fluor 647 and FITC by measuring the mean signal intensity in a defined area of 88 nm^2^. A total of 62 images were captured for each sample from 2 wells from 2 biological repeats. Images shown in Fig. 4 were deconvolved in Icy using the EpiDemic Plugin, and a maximum intensity projection was made in ImageJ.

## Supplementary Material

Supplemental file 1
