# Supplementary material for: A Biosynthetic Platform for Antimalarial Drug Discovery
Source: Antimicrob Agents Chemother. 2020 Apr 21;64(5):e02129-19. doi: 10.1128/AAC.02129-19 (PMC7179595; doi:10.1128/AAC.02129-19)
Supplement: Supplemental file 1 [file AAC.02129-19-s0001.pdf]

Supplemental Material for

A biosynthetic platform for antimalarial drug discovery

Mark D. Wilkinson[1], Hung-En Lai[2], Paul S. Freemont[2*] and Jake Baum[3*]

[1]Department of Chemistry, Imperial College London, UK

[2]Department of Infectious Diseases, Faculty of Medicine, Imperial College London, UK

[3]Department of Life Sciences, Imperial College London, UK

*Corresponding authors:

Jake Baum, Email: jake.baum@imperial.ac.uk

Paul S. Freemont, Email: p.freemont@imperial.ac.uk

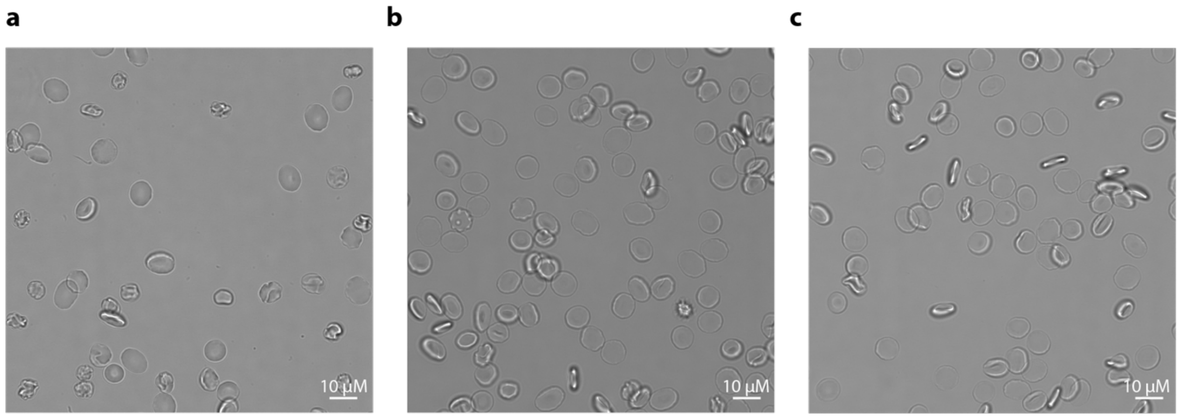

Fig. S1: Violacein does not affect red blood cell viability at the maximum concentration used in these assays.

a DMSO-treated red blood cell control

b Red blood cells treated with 2 µM Vio-Sigma.

c Red blood cells treated with 2 µM Vio-Biosyn.

The scale bar shown on each image is 10 µm.

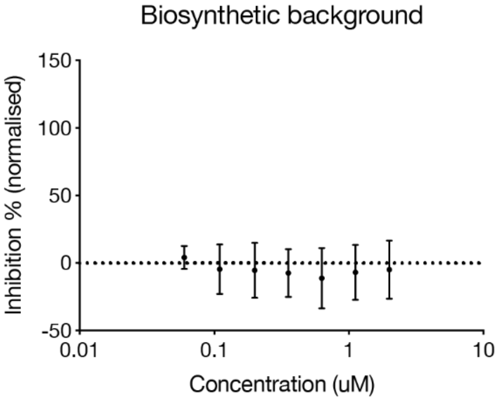

Fig. S2: Extract of *E. coli* cells without the violacein biosynthetic genes tested against *P. falciparum* 3D7 wild type parasites. The concentration of extract was adjusted to correspond to that of violacein-containing extract by normalising to the same wet cell mass. Data shown is of three technical replicates.

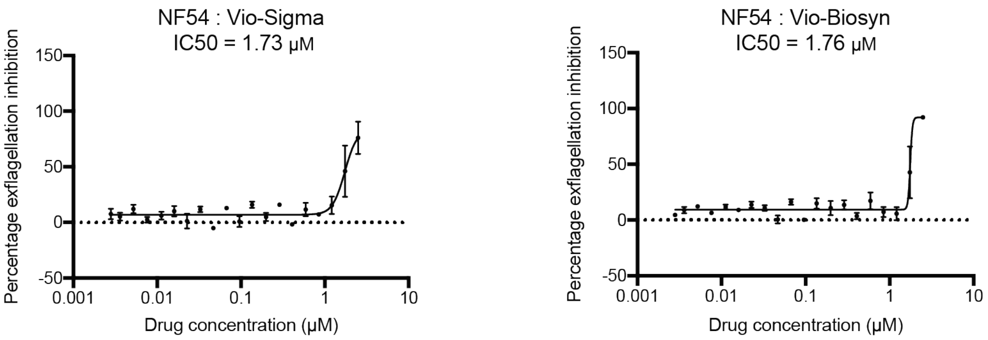

Fig. S3: Violacein and biosynthetic violacein inhibit exflagellation with similar IC50 values.

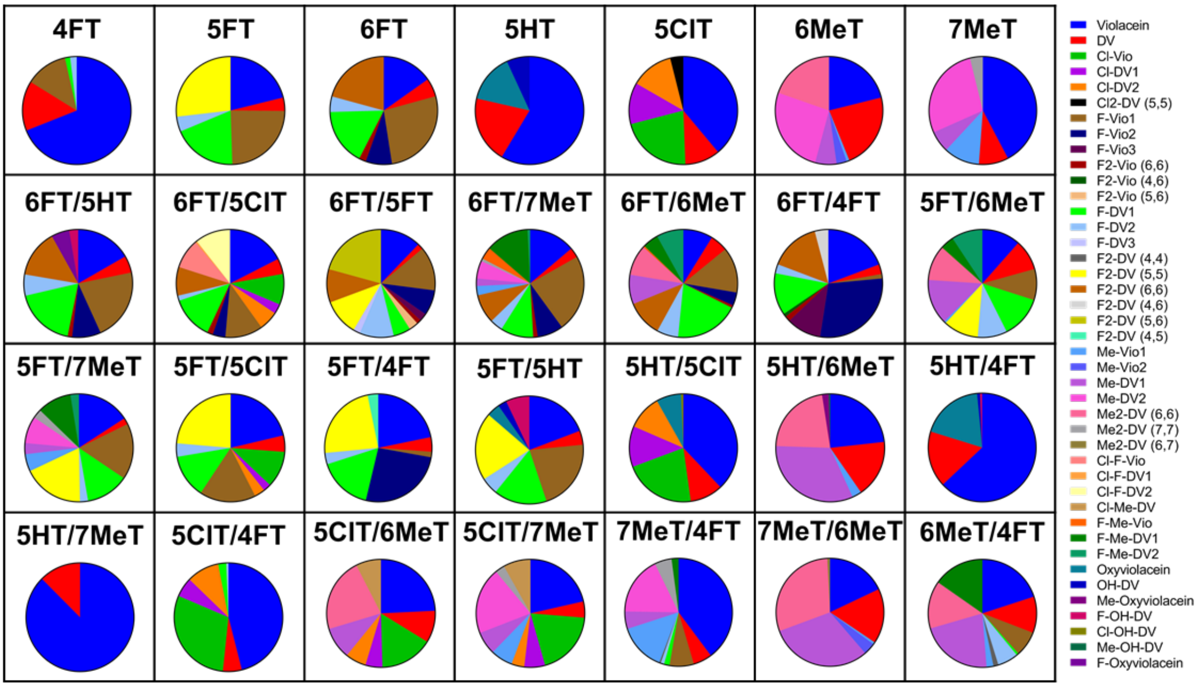

Fig. S4: Composition of the violacein derivatives used in the high-throughput single-dose screen. Figure adapted with permission from BioRxiv preprint (1) (https://www.biorxiv.org/content/10.1101/202523v2).

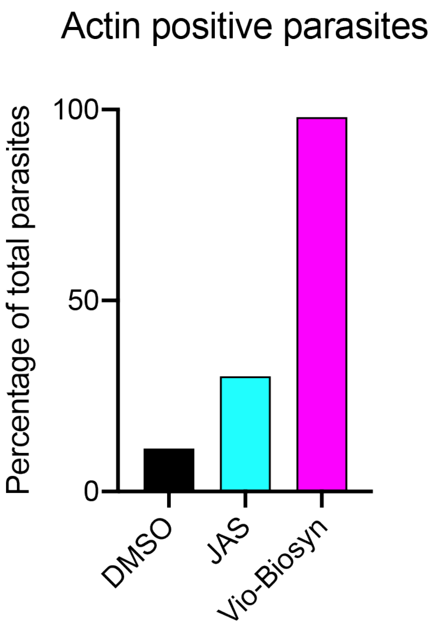

Fig. S5: Flow cytometry analysis of fixed 3D7 parasites (100 000 cells sorted). Parasitemia was estimated from an average of the nucleus (DAPI) and cytoplasm (GFP) positive parasites. Actin positive parasites were estimated as a percentage of red-positive cells over total parasitemia.

References:

1. Angrisano F, Riglar DT, Sturm A, Volz JC, Delves MJ, Zuccala ES, Turnbull L, Dekiwadia C, Olshina MA, Marapana DS, Wong W, Mollard V, Bradin CH, Tonkin CJ, Gunning PW, Ralph SA, Whitchurch CB, Sinden RE, Cowman AF, McFadden GI, Baum J. 2012. Spatial Localisation of Actin Filaments across Developmental Stages of the Malaria Parasite. PLoS One 7:e32188.
